# Talking about the hypothetical future: Serious illness communication for residents living with dementia in long-term care homes - An integrative review

**DOI:** 10.1177/26323524261462628

**Published:** 2026-06-25

**Authors:** Elizabeth Wojtowicz, Marie-Lee Yous, Pamela Baxter, Sharon Kaasalainen

**Affiliations:** 1 School of Nursing, 3710 McMaster University, Hamilton, ON, Canada

**Keywords:** serious illness communication, dementia, long-term care homes

## Abstract

**Background:**

In long-term care (LTC) homes, residents living with dementia frequently experience serious illness communication that is crisis-initiated and oriented to institutional documentation (e.g., transfer and resuscitation orders), rather than iterative, values-based discussions aligned with a palliative approach and substitute decision-making frameworks. Unpaid care partners often make high-stakes decisions with limited preparation, and residents are inconsistently included.

**Objectives:**

To explore how serious illness communication occurs with residents living with dementia, unpaid care partners, and healthcare providers in LTC, and to identify practice-relevant communication strategies and contextual factors applicable to clinical practice.

**Methods:**

An integrative review following Toronto and Remington’s six-stage methodology included 31 high-relevance studies (qualitative, quantitative, mixed methods, reviews, theoretical) published from 2015 to 2025 on serious illness, goals-of-care, or end-of-life communication in LTC dementia care. Directed content analysis was guided by Tarbi et al.’s basic science of communication in serious illness (lexical, non-lexical, contextual elements, and outcomes).

**Results:**

Serious illness communication was predominantly biomedical and documentation-focused, often occurring at admission or during crises and directed mainly to unpaid care partners, with limited resident involvement. Lexical practices such as clear, jargon-free information, explicit invitations to discuss “what matters most,” and early conversations about hypothetical future scenarios enhanced trust, preparedness, and alignment of care with resident values. Non-lexical elements (tone, eye contact, pacing, use of silence) shaped perceived empathy but were seldom explicitly addressed by interventions.

**Conclusions:**

For LTC healthcare providers, embedding earlier, iterative serious illness communication, explicitly involving residents where possible, and cultivating both lexical and non-lexical skills are key to achieving relationship-centred, legally compliant, and goal-concordant palliative approaches to care.​

## Plain language summary

This review looked at how people talk about serious illness and end-of-life care with residents living with dementia in long-term care (LTC) homes, and how these conversations affect care decisions. Serious illness communication includes discussions about what matters most to residents as their dementia progresses, what kinds of treatments they would or would not want, and how unpaid care partners and healthcare providers can prepare for future changes and dying.

The review found that these conversations in LTC usually happen late in the illness, often during admission to an LTC home or during a crisis and focus mainly on medical decisions or paperwork (for example, hospital transfer forms or resuscitation status). Residents with dementia are often not included directly and most conversations are held with unpaid care partners, who can feel unprepared or distressed. How healthcare providers communicate (by tone of voice, eye contact, body language, and use of silence) can strongly influence whether families feel heard, respected, and able to trust the team.

The way LTC homes are organized also shapes these conversations. Staffing levels, continuity of staff, time pressures, leadership support, and charting systems all influence whether serious illness discussions are ongoing and person-centred, or brief, checklist-style tasks. When communication works well, unpaid care partners report better understanding of what to expect, more confidence in making decisions, and care that is better aligned with the resident’s values near the end-of-life.

## Background

Dementia is a serious life-limiting illness characterized by progressive decline in cognitive functions such as memory, language, and reasoning.^1^ In long-term care (LTC) homes (also known as nursing homes, care homes, residential care facilities, and homes for the aged) dementia creates substantial communication barriers during serious illness decision-making.^2–5^ This affects residents living with dementia, their unpaid care partners (i.e. family, friends, substitute decision-makers or legal healthcare proxies), and healthcare providers including nurses, personal care workers, nurse practitioners, and physicians.^6–8^ In countries with established LTC systems, these barriers are compounded by high levels of multimorbidity, frailty, and polypharmacy among residents, making the quality and timing of serious illness communication particularly consequential for care trajectories and treatment-related burden.^9^

As dementia progresses, cognitive decline impairs instrumental activities of daily living, including financial and health-related decision-making, often years before a formal diagnosis is made.^10,11^ These changes gradually shift responsibility for everyday and serious medical decisions to unpaid care partners, increasing the risk of decisions that are misaligned with the person’s values, particularly near the end-of-life when treatment choices become more complex and burdensome.^12–14^ Serious illness communication addresses this through purposeful conversations and exchanges of information about the nature, progression, and management of serious health conditions between residents, unpaid care partners, and healthcare providers.^15,16^ However, serious illness communication is not limited to the dissemination of information. Relational, emotional, and contextual factors are central to how residents and unpaid care partners understand prognosis, weigh options, and cope with uncertainty, as highlighted in critical care and serious illness communication literature on the potential harms of “information overload”.^17^

Early and frequent serious illness communication enhances care experiences and aligns decisions with resident values.^14,18^ It also reduces unplanned healthcare encounters, such as emergency department visits and hospital admissions, near the end-of-life.^14,19,20^ In LTC, serious illness communication occurs alongside formal processes such as advance care planning (ACP) and substitute decision-making frameworks. While ACP aims to take place earlier in the disease trajectory, serious illness communication in LTC typically focuses on imminent or hypothetical health crises, and on translating existing values statements into concrete plans for hospitalization, treatments, and comfort-focused care.^21^ These conversations are especially critical upon move-in to a LTC home, helping unpaid care partners understand the disease trajectory for informed future substitute decisions.^16,22–24^

### Purpose

The purpose of this integrative review is to explore how serious illness communication occurs with residents living with dementia, their unpaid care partners and healthcare providers in LTC homes internationally. Previous reviews have examined ACP and end-of-life care for people living with dementia across community, hospital, and LTC settings, often focusing on documentation rates, completion of advance directives, or intervention effectiveness on health service use and caregiver outcomes.^21,25,26^ However, these reviews have paid less attention to the micro-processes of serious illness communication within LTC homes, including how conversations are initiated, which lexical and non-lexical practices are used, how contextual factors such as staffing, policies, and culture shape interactions, and how these processes unfold over time in established LTC systems.^16^ This integrative review addresses this gap by synthesizing qualitative, quantitative, mixed-methods, and theoretical work using Tarbi et al.’s serious illness communication framework, thereby providing a communication process-oriented understanding relevant to bedside practice and intervention design.^27^

Despite recognition of the importance of serious illness communication, its adoption remains limited in these settings. Barriers such as unpaid care partners’ misunderstanding of treatment limitations, poor acceptance of prognosis by residents and their unpaid care partners, insufficient healthcare provider training about communication in serious illness, time constraints for healthcare providers to have conversations, lack of documentation by all healthcare providers, and reluctance to communicate among all parties impede effective engagement.^14,28^ Given the complexities of cognitive impairment and unpaid care partner involvement in decision-making, it is essential to explore discrete communication elements to understand barriers and facilitators from the perspectives of residents and unpaid care partners.^2,28^

To explore how serious illness communication occurs with residents living with dementia in LTC homes and their unpaid care partners, Tarbi et al.’s (2022) basic science of communication in serious illness framework has been applied to identify and analyze a) key serious illness communication content used in LTC homes, b) contextual factors influencing communication effectiveness, and c) impacts of the communication content and contextual factors on outcomes for residents, unpaid care partners, and healthcare providers.

### Conceptual framework - Basic science of communication in serious illness

**Figure 1.**
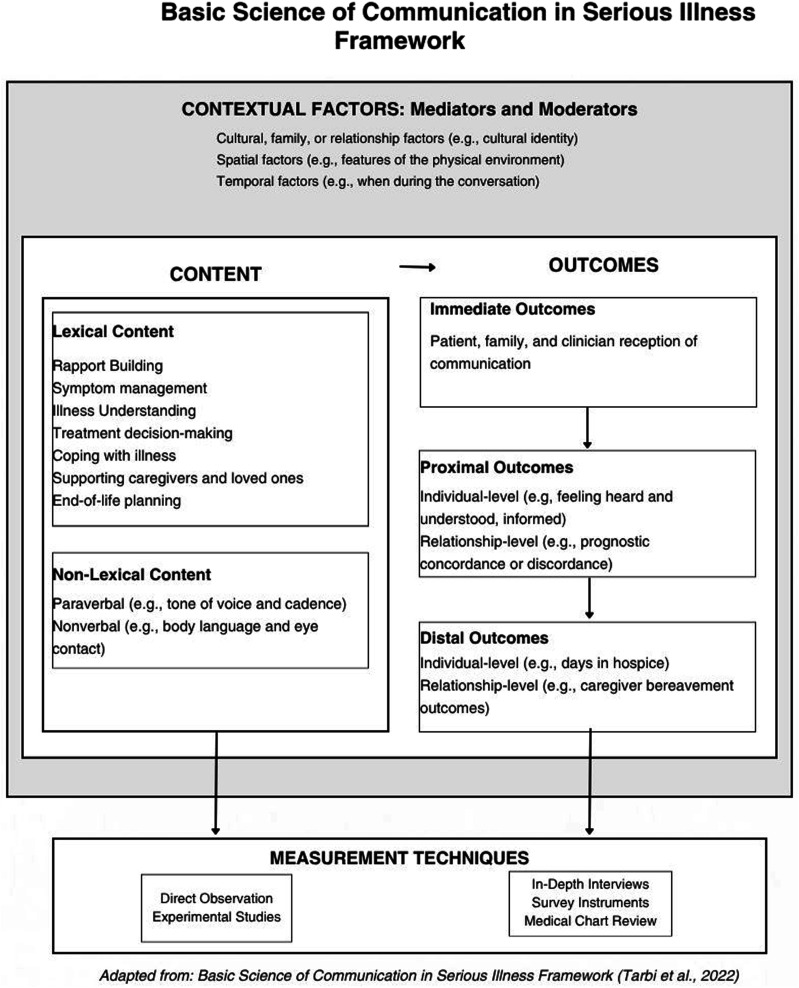
Conceptual framework: Basic science of communication in serious illness.

The overarching aim of Tarbi et al.’s^16^ framework of a basic science of communication in serious illness (see Figure – Basic Science of Communication in Serious Illness) is to inform evidence-based, targeted communication strategies that alleviate suffering, align care with goals, and reduce disparities in serious illness patient populations.^16,29^ The framework emphasizes discrete communication elements: lexical content (i.e. spoken or written elements such as words used during communication or information provided), non-lexical content (i.e. tone of voice, facial expressions, and body language), contextual factors (i.e. participants, timing, and location), and outcomes associated with the content and context of communication in serious illness (i.e. emotional impact, perceived communication quality, decisional preparedness, clinical actions, and health system impacts) (Figure 1).^16^

This review applies Tarbi et al.’s^16^ basic science framework to systematically understand the specific communication processes within the LTC home context and their outcomes for residents and unpaid care partners. By focusing on the ‘how’ of serious illness communication in dementia care, exploring communication content, contextual factors, and their impacts on outcomes, this review fills a gap in the literature that explores further then identifying barriers or describing broad communication concepts.^16^

## Method

An integrative review following Toronto and Remington’s six-stage methodology was conducted to synthesize qualitative, quantitative, mixed-methods, review, and theoretical studies on serious illness communication in LTC.^27^ The review focused on residents living with dementia, unpaid care partners, and healthcare providers in LTC homes in countries with established LTC structures. Reporting follows the PRISMA 2020 guidelines for systematic reviews, adapted for integrative review designs, in accordance with EQUATOR Network recommendations for transparent reporting of review methods and findings.^30^ No protocol for this integrative review was prospectively registered.

Integrative reviews aim to inform evidence-based practice by providing healthcare providers with synthesized evidence to guide clinical decision-making and improve patient care.^31^ This methodology was chosen over other types of review methods (e.g., systematic review) because it supports the integration of diverse research designs, enabling an exploration of the complex process of serious illness communication with residents living with dementia in LTC homes.^31^ The current state of the evidence, its quality, and gaps in the literature were identified, followed by implications for clinical practice, education, policy, and research.^31,32^

### Structured literature search

A comprehensive electronic search was performed for literature published between January 1, 2015, and August 8, 2025. The start date was selected to align with the dementia human rights movement’s influence on discourse toward the empowerment and social inclusion of people living with dementia, which came in parallel with the World Health Assembly’s 2014 resolution calling for the integration of palliative care across all levels of health systems.^33–35^

Six databases were searched: CINAHL, AgeLine, Emcare, Google Scholar, Medline, and PsycINFO. The search strategy was developed and refined over two consultations with a Health Sciences librarian to optimize database selection, keywords, and search limits. The Population-Concept-Context (PCC) framework guided term selection, focusing on residents living with dementia (Population), communication in serious illness (Concept), and LTC homes (Context).^36^ Core keywords and MeSH terms were combined using Boolean operators to maximize retrieval (e.g., “dementia AND long-term care homes AND communication in serious illness”) with synonyms for each key term included via the OR operator. The complete search strategy is presented in Table - Literature Search Strategy .

### Inclusion and Exclusion Criteria

Eligible studies included qualitative, quantitative, mixed methods, systematic reviews, theoretical research, and grey literature (dissertations) that: a) focused on communication in serious illness processes involving residents living with dementia in LTC homes and their unpaid care partners; b) clearly described research questions, the context, study samples, and data collection and analysis methods; and c) were published in English with full-text available. The excluded studies were a) those involving residents outside LTC settings (e.g., hospitals, assisted living, home care); b) research not focused on communication processes related to serious illness (e.g., intervention implementation, symptom management, broad palliative care); and c) studies without sufficient descriptive or theoretical detail. The detailed criteria are summarized in Table - Inclusion and Exclusion Criteria.

### Screening and selection process

A staged review was undertaken with references managed using Covidence software, which facilitated duplicate removal and organization of results.^37^ After de-duplication, 465 unique records were identified from 688 citations. The database search identified 413 references, with a further 52 sources identified through reference list citation searching (n=44) hand-searching (n=4) and consultation with three subject matter experts, one each in dementia care, long-term care and palliative approach to care (n=4). Titles and abstracts were screened, and 132 studies were excluded from the review. Full texts of 110 articles were assessed, with 64 articles excluded for irrelevance or failure to meet criteria (29 not about process; 15 not about communication in serious illness; 12 outside of LTC; eight not about dementia). A total of 46 articles were included in the data evaluation. Screening and selection were performed by the author, with consultation and consensus sought with the fourth author (S.K.) for unclear cases. The detailed process of selection is presented in the PRISMA diagram (Figure – PRISMA diagram: Integrative review).

### Data evaluation

In an integrative review, relevance is pivotal when the aim is to synthesize evidence that directly influences care practices, and strict adherence to methodological rigour may exclude insights from studies that do not meet traditional standards, but provide valuable, context-sensitive findings.^38^

### Relevance appraisal

The studies in this review were scored on their relevance and informational value to serious illness communication in dementia care and their contribution to understanding the core concepts of the research questions. Studies of high relevance directly addressed the research question or core concepts, provided empirical data or systematic review findings on communication processes, and included: a) content and context of communication, b) primary perspectives from residents, unpaid care partners, or healthcare providers in LTC dementia contexts, and c) specifically focused on communication processes, timing, or barriers/facilitators in serious illness or end-of-life discussions. Studies of moderate relevance discussed related topics (such as advance care planning, ethical concerns, issue-specific care, or palliative care in dementia), or addressed communication in LTC but included broader populations or related illnesses beyond dementia. Low relevance studies did not include LTC settings and covered general communication or healthcare topics without specific relevance to serious illness communication in dementia (such as dementia epidemiology, pharmacological treatment, day-to-day care processes, or non-communication aspects of LTC).

### Critical appraisal

This integrative review included diverse methodologies, and critical appraisal was performed using design-specific tools addressing the quality of studies. These included the following tools: Delphi,^39^ mixed methods,^40^ qualitative,^41^ analytical cross-sectional and cohort,^42,43^ systematic reviews,^44^ and theoretical research.^31^

Consistent with the prioritization of relevance over methodological rigour in integrative reviews,^31^low-quality and low or moderate-relevance studies were excluded, leaving one high-relevance/moderate-quality study^45^ and 30 high-quality/high-relevance studies for synthesis.

### Data analysis and synthesis

Directed content analysis was used for data analysis in this study. In line with Hsieh and Shannon’s approach,^46^ a deductive codebook was developed based on Tarbi et al.’s^16^ conceptual framework for a basic science of communication in serious illness, providing a structured coding scheme for the analysis. Initial coding was performed by the first author, with a subset independently reviewed by the fourth author (S.K.) to ensure reliability.

A data extraction matrix was constructed to organize key information from the studies, including study design, objectives, characteristics of study participants and setting, lexical and non-lexical content, contextual communication factors, and outcomes measured (Supplemental File – Characteristics of Studies). The matrix facilitated a systematic comparison of data across studies, allowing directed analysis to remain anchored in the predefined coding categories while permitting the construction of additional relevant themes.^46^

Following Hsieh and Shannon’s^46^ principles, the synthesis phase involved the organization and integration of extracted data into framework categories that reflected the core elements of communication in serious illness, such as resident and unpaid care partner involvement, timing, and communication strategies used. The process allowed exploration of relationships and patterns across studies, enabling a deeper understanding of the dynamic interplay between serious illness communication content, communication methods, and contextual influences. Data from diverse designs (qualitative, quantitative, mixed-methods, reviews, theoretical papers) were synthesized using a convergent integrated approach. Quantitative findings (e.g., changes in family preparedness scores and hospitalization rates) were transformed into qualitative descriptors (e.g., ‘increased family preparedness’, ‘reduced hospital transfers’) before being integrated with qualitative themes mapped to Tarbi et al.’s lexical, non-lexical, contextual, and outcome domains. This process followed JBI recommendations for data extraction and transformation in convergent integrated mixed-methods reviews.^47^ Consistent verification processes, including constant comparison, supported trustworthiness of interpretations.^46^ The resulting synthesis reflects the nuanced and multifaceted nature of communication in serious illness with residents living with dementia in LTC homes and its impact on relationship-centred care and decision-making.

## Results

This review synthesized 31 studies on communication in serious illness involving residents living with dementia and their unpaid care partners in LTC homes. The following section outlines key characteristics of included studies and findings. Most studies used qualitative designs featuring semi-structured interviews. Findings highlight communication processes, contextual factors, and the influence on residents, unpaid care partners, and healthcare provider outcomes, providing a comprehensive understanding of serious illness communication in these settings.

### Measurement techniques

The basic science of communication in serious illness framework highlights a suite of complementary measurement techniques to link discrete lexical, non-lexical, and contextual elements with outcomes. These include direct observation to measure what is said and how it is said, in-depth interviews to capture emotional and cognitive responses, and quantitative surveys and chart reviews to assess outcomes such as perceived communication quality, goal-concordant care, and health service use.^16^ Additionally, experimental and virtual reality designs, physiological measures, and AI-assisted analysis are suggested as more rigorous study pathways to consider^16^

Across the 31 included studies, serious illness communication was assessed using a range of qualitative and quantitative methods, including semi-structured interviews (n=12), structured questionnaires (n=6), chart review(n=4), direct observation (n=4), and focus groups (n=3). Semi-structured interviews were the most common method to explore how residents, unpaid care partners, and LTC staff experienced and interpreted communication around prognosis, goals of care, and end-of-life decisions (e.g., 48–50). Quantitative tools such as the Quality of Communication questionnaire and family preparedness measures were used less frequently but provided complementary data on perceived quality and outcomes of communication (e.g., 51. No studies included physiological measurement, experimental use of VR, or AI-assisted coding, indicating missed opportunities to robustly connect discrete elements with multi-level outcomes.

### Study design

Fifteen studies (n=15) used a qualitative design, including descriptive, interpretive, and constructivist grounded theory^13,50,52–64^; eight studies (n=8) were quantitative, including cohort, cross-sectional, and cluster randomized control trials^51,65–71^; four studies (n=4) were reviews^45,72–74^; two studies (n=2) were theoretical research, including conceptual model development and situation-specific theory^75,76^; and there is one study each of Delphi design and mixed methods.^26,77^

### Participants

Most studies primarily sampled LTC staff, particularly nurses and personal support workers, with fewer studies focusing on unpaid care partners or residents. Staff perspectives dominated 20 of the 31 studies, whereas only three studies focused mainly on unpaid care partners and two on residents themselves (e.g., 49, 50 and 57). Mixed samples combining residents, unpaid care partners, and staff were used in several studies to examine communication processes across roles (e.g., 63 and 68).

### Settings – Countries

Most of the included studies were conducted in Western, high-income countries, including the United Kingdom, other European nations, the United States, Canada, and Australia. This concentration reflects LTC systems with broadly similar regulatory and funding structures and has implications for the transferability of findings to non-Western or low-resource settings (e.g., 45, 49 and 64).

### Findings

**Figure 2.**
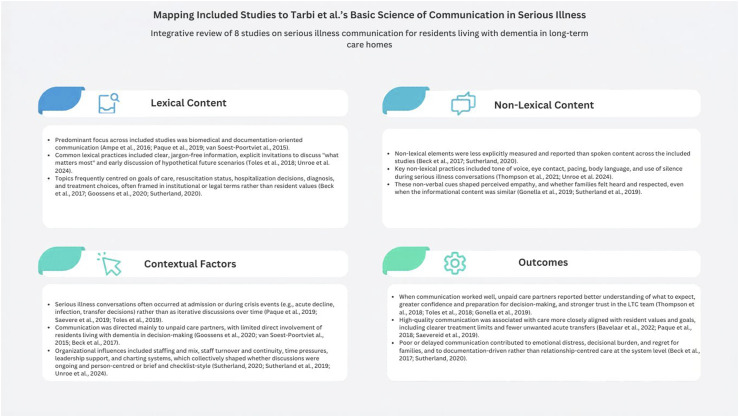
Mapping Tarbi et al.’s (2022) domains to findings.

When mapped onto Tarbi et al.’s framework, almost all studies contributed data on lexical content, many described contextual factors, and fewer examined non-lexical elements or outcomes in depth.^16^ Lexical practices such as providing clear information, explaining prognosis, and discussing hospitalization and resuscitation decisions were common focal points (e.g., 65, 70 and 71). Non-lexical features such as tone, eye contact, and pacing were often acknowledged as important but were rarely defined or measured systematically (e.g., 50 and 67). See Figure – Domain Mapping (Figure 2).

### Lexical content

Across the included studies, lexical content focused on how healthcare providers, residents, and unpaid care partners used words to build trust, clarify illness and prognosis, and navigate communication in serious illness and treatment decisions.^64,75,78,79^ Communication strategies such as rapport building, active listening, and clear, jargon-free explanations are consistently linked to residents’ and unpaid care partners’ sense of being heard, respected, and supported in decision-making.^45,58,60,74^ Healthcare providers who explicitly invited questions, acknowledged emotions, and explored “what matters most” facilitated a more open discussion of goals of care and end-of-life preferences in the context of dementia.^56,70,80,81^

Several studies described how trust and ongoing relationships shaped the conditions for serious illness communication in long-term care homes. Healthcare providers reported working to create a familiar, collaborative atmosphere in which residents living with dementia and unpaid care partners felt able to raise questions and talk about ‘what if’ scenarios related to deterioration or end-of-life.^48,64^ Unpaid care partners valued honest, consistent information and person-centred communication from staff, and described feeling more comfortable expressing emotions when interactions were not limited to biomedical topics.^48,52,79^ These relational features were reported as reducing anxiety and laying groundwork for more sensitive discussions about functional decline, symptom changes, and end-of-life care.^50,64^

### Non-lexical content

Non-lexical features, including tone of voice, facial expressions, eye contact, and body language, play a critical role in shaping how verbal messages are interpreted and experienced.^53,63,76^ Calm, steady tone, warm facial expressions, and sustained, appropriate eye contact were perceived by unpaid care partners as signals of compassion, safety, and willingness to engage in difficult topics, enabling the disclosure of emotions and questions about prognosis and serious illness trajectories.^13,50,53,57,62,75,78^ In contrast, rushed cadence, flat affect, or avoidant gaze communicate discomfort or disinterest, discouraging further conversation and sometimes reinforcing unpaid care partners’ reluctance to raise end-of-life concerns.^64^

Studies have also highlighted that non-lexical cues operate as a feedback loop in serious illness encounters. Providers who noticed residents’ or unpaid care partners’ emotional expressions, changes in posture, or withdrawal from eye contact adjusted pacing, allowed silence, or softened their tone to maintain connection and regulate distress.^64^ This adaptive use of nonverbal behaviours supported resident-centred dialogue by giving space for reflection, grief, and meaning-making, especially when discussing prognosis, decline, or impending death in the context of dementia.^45,53,56,74^

### Contextual factors

Communication was deeply embedded in contextual factors, including who was involved, when and where conversations occurred, and how organizational structures shaped opportunities for serious illness communication and end-of-life discussions.^16^ At the interpersonal level, long-standing relationships and familiarity between healthcare providers, residents, and unpaid care partners facilitated more candid and iterative conversations, whereas high turnover, limited continuity, and ambiguous role expectations undermined trust and clarity regarding who was responsible for leading serious illness communication.^45,50,56,57,59,63,66,67^

Across the review, serious illness communication in long-term care homes was predominantly conducted with unpaid care partners, with limited direct involvement of residents living with dementia. Healthcare providers frequently cited concerns about cognitive capacity, communication difficulties, and emotional burden as reasons for focusing on care partners, although some studies reported that residents could contribute meaningfully to discussions about comfort, place of care, and priorities when conversations occurred early in the disease trajectory.^48,63,68^

Serious illness conversations were most commonly documented at admission or during acute clinical changes, such as infections, falls, or marked functional decline.^70,71^ Few studies described communication as an iterative process. Instead, discussions about goals of care and end-of-life preferences were often clustered around discrete events.^51,65^

Several studies described a tension between ‘living in the moment’ and planning for the future among residents living with dementia and their unpaid care partners. Residents often preferred to focus on day-to-day concerns and avoided explicit discussion of an uncertain future, whereas unpaid care partners described both anxiety about potential decline and a desire for more information.^50,52^ In some studies, residents and unpaid care partners reported waiting for healthcare providers to invite discussion about what might happen next, but such invitations were not always offered.^48,64^

Few studies provided detailed accounts of residents’ and unpaid care partners’ cultural, racialized, or linguistic backgrounds, limiting inferences about how culture shapes serious illness communication in long-term care. Nonetheless, available descriptions suggest that norms around familial responsibility, truth-telling, and deference to healthcare providers can influence who is invited into conversations, how information is framed, and whether residents’ preferences are elicited directly.^13,53,55,58,75,80^

A number of studies demonstrated how organizational and system-level factors often constrain the enactment of high-quality communication, despite individual willingness and skill.^49,55,60,69,75,76^ Heavy workloads, staffing shortages, and task-oriented routines in LTC homes limited the time available for serious illness conversations and in-depth end-of-life discussions. This resulted in care conferences that were either not attended by key decision-makers or that focused on immediate care needs rather than future planning and residents’ values.^64,67,68,73^ Documentation systems, regulatory pressures, and professional boundaries sometimes placed a greater focus on biomedical information and risk management versus the relational aspects of communication, reinforcing the tension between objectivity and the social-emotional labour required for serious illness conversations.^50,55,64,65,67,73^

### Outcomes of communication in serious illness

Outcomes associated with lexical, non-lexical, and contextual elements of communication were described at the immediate, proximal, and distal levels.^16^ Immediate outcomes included residents and unpaid care partners who experienced validating tone, empathic responses, and opportunities to express emotions reported feeling more prepared and less isolated when facing symptom deterioration and end-of-life transitions.^45,50,58,67,78^ Proximal outcomes involved honest, compassionate, and person-centred communication that reduced anxiety, supported anticipatory grief, and fostered acceptance among unpaid care partners, whereas avoidance of prognosis or serious illness conversations contributed to ongoing uncertainty, regret, and moral distress after the resident’s death.^53,64,75^ Distal outcomes reflect the extent to which communication supports goal-concordant care and the efficient use of health services.^49,56,57,71,75,80,82^

When communication in serious illness and end-of-life discussions were timely, iterative, and grounded in residents’ values, care plans were more often aligned with comfort-focused preferences, reduced unwanted transfers, and clarified treatment and resuscitation limits.^55,56,59,70,75,82^ Conversely, fragmented or delayed communication is associated with crisis-driven decisions, potentially non-beneficial hospitalizations, and missed opportunities to align care with residents’ and unpaid care partners’ goals, highlighting the centrality of serious illness communication to quality of life, bereavement experiences, and healthcare system performance in LTC dementia care.^63,67,73,77^

## Discussion

This integrative review used Tarbi et al.’s framework to guide a critical synthesis of existing evidence related to the processes and content of serious illness communication in LTC homes involving residents living with dementia. Drawing on a range of empirical studies, it is evident that effective communication is essential for aligning care with resident values and preferences, supporting unpaid care partner decision-making, and improving end-of-life outcomes.^75,78,79^ The synthesis aligns with international reports that serious illness and end-of-life discussions in dementia often occur late, are oriented to documentation, and rarely include residents directly.^21,25,26^ Despite the recognized significance of these processes, the literature reveals persistent challenges related to the complexities of cognitive decline, inconsistent practices, and organizational barriers, which collectively hinder the delivery of timely, relationship-centred serious illness communication.^28,67,77,83^ This discussion explores the issues further, considering implications for practice, education, policy, and future research, with the aim of advancing understanding and implementation of meaningful serious illness communication in LTC homes.

This integrative review highlights the ways in which residents living with dementia and their unpaid care partners engage in conversations about hypothetical future scenarios and plan for care over time. By organizing findings within Tarbi et al.’s framework, specific factors are identified that may mediate trust, decisional preparedness, and goal-concordant care in long-term care homes. The findings reinforce arguments that serious illness communication cannot be reduced to the provision of prognostic information or completion of forms. Communication that focuses narrowly on information transfer risks overwhelming families and neglects the emotional, relational, and cultural dimensions of decision-making that are central to a palliative approach to care.^17^

Study findings suggest that timing is central to serious illness communication in LTC dementia care. Conversations were most helpful when they occurred early enough to include residents where possible, but flexibly enough to account for adjustment to diagnosis, transition into LTC, and changing decisional capacity over time.^48,50,63^ In contrast, crisis-driven discussions or late end-of-life meetings often left unpaid care partners feeling unprepared and distressed.^50,51^

Across studies, trust emerged as a foundational condition for discussing hypothetical future scenarios, decline, and end-of-life care. Unpaid care partners were more willing to raise concerns, express emotion, and participate in values-based discussions when they experienced staff as consistently present, honest, and emotionally available.^48,64,79,84^ Where relationships were weak, or staff turnover was high, unpaid care partners described feeling like outsiders and tended to avoid serious illness conversations even when they wanted more information.^49,50^

Resident involvement was limited in most studies, despite evidence that many people living with dementia can contribute meaningfully to discussions about comfort, place of care, and what matters most at different points in the illness trajectory.^48,63^ Healthcare providers frequently cited concerns about cognitive capacity, communication difficulties, and emotional burden as reasons for focusing on unpaid care partners. These patterns raise important questions about how personhood and relational autonomy are enacted in long-term care dementia communication.^67^ While concerns about decisional capacity are legitimate, excluding residents by default risks reinforcing paternalism and may overlook opportunities for shared or supported decision-making.^48,68^

The review reinforces that serious illness communication is not a single event but an evolving process shaped by clinical change and family readiness.^53,56^ When conversations were limited to admission or acute crises, unpaid care partners often felt unprepared and described decisions as rushed or reactive.^50,51^ By contrast, studies that described early and revisited discussions suggested greater decisional preparedness and more alignment between care and resident values.^65,71^

### Implications for clinical practice

The findings of this integrative review are directly relevant to clinical practice in long-term care homes. Effective serious illness communication in this context requires combining clear, concrete discussion of prognosis and treatment options with relational work that acknowledges emotion, supports anticipatory grief, and maintains personhood.^48,50,64^ Organizational conditions such as staffing, continuity, time, and role clarity are therefore integral to communication quality, rather than background factors.^49,62^

Integrating values-based serious illness communication, by focusing on “what matters most” to residents and unpaid care partners, with shared decision-making about goals offers an opportunity to align decisions with residents’ priorities, improve decisional confidence, and reduce treatment burden near the end-of-life.^11,53^ Embedding these conversations into routine LTC practice, rather than limiting them to crisis events or admission paperwork, may therefore support both goal-concordant care and the broader culture change that is underway in many LTC homes internationally.^85^

### Implications for education

Educationally, the review underscores the need for dementia-specific serious illness communication training that addresses both verbal and non-verbal elements of difficult conversations.^49,67,79^ Programs that integrate biomedical content with relationship-centred skills and attention to cognitive change may better prepare healthcare providers to navigate complex communication in LTC dementia care.^67^

### Implications for policy

At the policy level, the findings suggest that serious illness communication should be recognised as core LTC work, supported by regulations and quality frameworks that enable time, continuity, and role clarity for these conversations.^49,71^ Policies that reduce reliance on crisis-driven decision-making and support earlier, iterative communication are likely to improve alignment between care and resident values.

### Implications for research

Future research should move beyond cataloguing barriers to testing and refining dementia-specific communication models in long-term care. Priorities include work that centres the voices of residents living with dementia, non-lexical communication, cultural and racialized contexts, and the mechanisms linking communication processes to outcomes that matter to residents, unpaid care partners, and healthcare providers.^16,50,64^

### Strengths and limitations

Tarbi et al.’s^16^ conceptual framework significantly enhanced the rigour and depth of this integrative review by providing a systematic approach to extracting and analyzing the discrete elements of serious illness communication within LTC homes. Specifically, the framework’s delineation of lexical content, non-lexical content, contextual factors, and outcomes enabled a focused examination of communication processes that are often complex and multifaceted in dementia care.^16^ This structured lens facilitated the identification of nuanced communication features that influence resident and unpaid care partner experiences, often overlooked in more generalized reviews.^46^ Importantly, applying the framework supported the synthesis of diverse study designs and methodologies, ensuring a comprehensive and conceptually coherent integration of findings.^31^ Consequently, the framework not only guided data organization and thematic development but also highlighted key gaps and potential targets for intervention, thereby advancing in the understanding of serious illness communication processes in this vulnerable population.

In addition to the framework elements, it became apparent that communication within LTC homes is shaped by broader, interacting factors that influence how care goals are discussed and enacted over time. These factors extend beyond information exchange to encompass relationship quality, emotional response management, alignment with values and preferences in decision-making, and organizational conditions that enable or hinder dialogue. Understanding this interplay provides a structure for examining how communication processes support relationship-centred and goal-concordant care in LTC dementia contexts.

Finally, the inclusion of high-relevance studies that capture primary perspectives from residents living with dementia, their unpaid care partners, and healthcare providers facilitated a nuanced and relationship-centred analysis of communication in serious illness in the LTC context.

Limitations include the small number of studies meaningfully addressing the non-lexical communication elements,^45,54,59,68,79^ and the lack of focus on outcomes in the quantitative studies, limiting the applicability to the research questions. The literature was dominated by healthcare provider perspectives, with considerably fewer studies capturing the direct experiences of residents living with dementia and their unpaid care partners, potentially constraining the relationship-centred focus.^86^ Additionally, few studies explicitly addressed how culture, migration history, and structural inequities may interact with long-term care policies and staffing to shape who is included in conversations, when they occur, and how prognosis is framed.^11,44,46,62^ Where the primary studies provided limited detail, this is explicitly acknowledged as a gap.

The reliance on secondary analysis means findings are shaped by the methodological rigour, sample characteristics, and context-specific details of existing studies, transferring any inherent gaps and biases into the synthesis.^31^ No protocol for this integrative review was prospectively registered on PROSPERO or another registry, which is acknowledged as a limitation. Additionally, restricting the search to English-language publications from the past 10 years may exclude relevant earlier or non-English studies. Finally, most included studies originate from Western, high-income countries (primarily the United Kingdom, Europe, United States, and Canada), limiting cultural applicability and relevance for non-Western, low-resource or low-income countries.

## Conclusion

Communication processes in LTC for residents with dementia are deeply relational, contextually influenced, and reliant on ongoing support, education, and engagement of residents living with dementia and unpaid care partners to achieve goal-concordant and compassionate care. Effective communication involves both the content - what is discussed, such as illness trajectory, goals of care, and emotional concerns - and context - including the timing, setting, and relational dynamics between residents, unpaid care partners, and healthcare providers. Conversations that integrate both medical and psychosocial topics, adapting to cognitive changes and communication challenges, foster trust and encourage shared decision-making. Timely and proactive conversations, rather than being reactive and crisis driven, enable the inclusion of resident preferences and unpaid care partner input to support quality of life, personhood, and dignity. Ultimately, improving both the content and context of communication in serious illness through education, policy, and future research remains essential to supporting the complex needs of residents living with dementia in LTC homes.

## Supplemental material

Supplemental material - Talking about the hypothetical future: Serious illness communication for residents living with dementia in long-term care homes - An integrative review Supplemental material for alking about the hypothetical future: Serious illness communication for residents living with dementia in long-term care homes - An integrative review by Elizabeth Wojtowicz, Marie-Lee Yous, Pamela Baxter, Sharon Kaasalainen in Palliative Care and Social Practice

Supplemental material - Talking about the hypothetical future: Serious illness communication for residents living with dementia in long-term care homes - An integrative review Supplemental material for alking about the hypothetical future: Serious illness communication for residents living with dementia in long-term care homes - An integrative review by Elizabeth Wojtowicz, Marie-Lee Yous, Pamela Baxter, Sharon Kaasalainen in Palliative Care and Social Practice

## Data Availability

All data generated or analysed during this study are included in this published article and its supplementary information files.*
