# Supplementary material for: Talking about the hypothetical future: Serious illness communication for residents living with dementia in long-term care homes - An integrative review
Source: Palliat Care Soc Pract. 2026 Jun 25;20:26323524261462628. doi: 10.1177/26323524261462628 (PMC13305509; doi:10.1177/26323524261462628)
Supplement: Supplemental material - Talking about the hypothetical future: Serious illness communication for residents living with dementia in long-term care homes - An integrative review [file sj-pdf-1-pcr-10.1177_26323524261462628.pdf]

Recommendations for Practice, Education, Policy, and Research

1. Foster relationship-centred care culture valuing personhood (Practice): Promote cultures that affirm personhood, agency, and psychosocial well-being of residents living with dementia, positioning unpaid care partners alongside healthcare providers (39, 40, 48).
2. Initiate early, flexible communication in serious illness post-dementia diagnosis (Practice): Begin communication in serious illness around dementia diagnosis, or shortly after moving into an LTC home, in a relationship-centred manner, adapting to readiness and comfort to preserve personhood and familial bonds (38, 53, 66).
3. Personalize communication in serious illness for fluctuating cognitive and decision-making capabilities (Research/Practice): Explore tailoring communication in serious illness to cognitive capacity, health literacy, culture, and preferences, developing models that support fluctuating and substitute decision-making (66).
4. Train healthcare providers in dementia-specific communication skills (Education): to build trust, elicit values, discuss prognosis, support shared decision-making, and tailor to cognitive capacity, health literacy, culture, and preferences (42, 52, 63, 67).
5. Embed dementia care education into LTC home practices (Practice/Education): Establish continuing education programs and support nursing leadership initiatives to embed serious illness communication into LTC home routines and ensure sustained engagement (60, 67).
6. Clarify healthcare provider roles in serious illness communication (Policy): Develop organizational policies clarifying nurse and personal care worker roles in serious illness communication that support interdisciplinary collaboration (39, 48, 60).
7. Investment in broad education initiatives (Education): To equip future and current healthcare providers with skills to engage in early and effective communication in serious illness discussions, educational frameworks and policies should embed gerontological competencies and serious illness communication training requirements into healthcare learner curricula (79, 81).
8. Promote national policies that support equity in advance dementia care (Policy): aiming to address systemic and racialized disparities, mandate early and ongoing communication in serious illness in practice, and fund public-facing education to increase understanding and uptake of communication in serious illness (44, 53, 74).
9. Design studies to explore optimal content and process of communication in serious illness conversations (Research): Include components on early initiation of communication in serious illness with residents living with dementia, how many conversations are feasible and how to prioritize topics, ideally while allowing flexibility to address readiness and comfort levels (66).
10. Develop or refine dementia-specific communication in serious illness practice models (Research): Investigate methods to effectively support decision-making with impaired capacity, incorporating concepts of relational autonomy and person-centred approaches, recognizing changes over time in patients’ preferences and decisional capacity (66).
