# Supplementary material for: Talking about the hypothetical future: Serious illness communication for residents living with dementia in long-term care homes - An integrative review
Source: Palliat Care Soc Pract. 2026 Jun 25;20:26323524261462628. doi: 10.1177/26323524261462628 (PMC13305509; doi:10.1177/26323524261462628)
Supplement: Supplemental material - Talking about the hypothetical future: Serious illness communication for residents living with dementia in long-term care homes - An integrative review [file sj-pdf-2-pcr-10.1177_26323524261462628.pdf]

Manuscript ID PCR-26-0022_Revised

Characteristics of Studies (mapped to Tarbi et al. (2022) framework)

Author	Year	Country	Study Focus	Study Design & Data Collection	Participants	Fran
Ampe	2017	Belgium	Influence of ‘we DECide’ communication intervention on advance care planning policy and practice in dementia care	Design: Mixed methods<br>Data Collection: Evaluated policy with ACP-audit, and involvement of residents/ families with ACP criteria and OPTION scale	N = 149 (N = 90 nursing home staff (management and clinical), N=13 residents with dementia took part in survey; N=16 family members, and N30 staff members were involved in 21 recorded ACP conversations)	**Lexi**<br>talk,<br>**Cont**<br>famil<br>socia<br>staff.<br>Temp<br>deter<br>Orga<br>boun<br>barri
Ampe	2016	Belgium	Advance care planning (ACP) policy vs practice in nursing home residents with dementia	Design: Observational cross-sectional<br>Data Collection: Data collected using three instruments: ACP audit, self-developed ACP criteria, and the OPTION instrument (evaluating resident/family involvement).	N = 153 Nursing home staff (managers, head nurses, nurses, auxiliaries, social workers, therapists, psychologists)	**Lexi**<br>pros/<br>**Cont**<br>highe<br>invol<br>Orga<br>based<br>clinic<br>Empl<br>over
Ashton	2016	U.K.	Experiences of advance care planning (ACP) among family caregivers for people with advanced dementia	Design: Qualitative Description<br>Data Collection: Semi-structured, in-depth interviews	N = 12 family caregivers (mainly spouses and adult children)	**Lexi**<br>treatr<br>**Non-**<br>disco<br>readi<br>**Cont**<br>unpre<br>influe<br>Temp<br>consi<br>Orga<br>delay<br>distre
Bavelaar	2022	Netherlands	Changes in advance care plans of nursing home residents with dementia following pneumonia	Design: Secondary analysis of a cluster-randomized controlled trial<br>Data Collection: Chart audit: comparing advance care plans before and after the first pneumonia episode and using generalized logistic linear mixed models to explore associations with changes	N = 429 residents with dementia who developed pneumonia, elderly care physicians	**Lexi**<br>prolo<br>90%<br>episo<br>Plan<br>artifi<br>**Cont**<br>incre<br>resid<br>Temp<br>sever

Beck	2017	U.K. (8), Europe (3), U.S.(2), Australia (1)	Healthcare Provider (HCP) Perspectives on ACP in Dementia	Design: Narrative Review<br>Data Collection: Systematic Review	N = 1305 participants across 14 included studies: N = 1305 healthcare professionals (HCPs) from various professional backgrounds.	**Lexi**<br>disco<br>futur<br>dyna<br>clear<br>**Cont**<br>famil<br>Temp<br>recor<br>delay<br>Orga<br>traini<br>respo<br>influ<br>and p
Beck	2017	UK	Knowledge, attitudes, beliefs and practice toward ACP for people with dementia	Design: Cross-sectional survey<br>Data Collection: Postal survey: A specific tool was developed for this clinical setting, informed by a previously validated instrument and the theory of planned behavior	N = 116 Nursing home managers	**Lexi**<br>care,<br>wishe<br>**Non**<br>listen<br>emot<br>**Cont**<br>subst<br>allied<br>Temp<br>deme<br>chang<br>Orga<br>clear
Daneau	2022	Canada (Quebec)	To develop a grounded theory on nurses' support of relatives making end-of-life decisions for residents living with advanced dementia in long-term care homes.	Design: Constructivist Grounded theory<br>Data Collection: Face-to-face semi-structured interviews, a sociodemographic questionnaire, and three end-of-life documents	N = 19 (N=9 nurses, N=10 relatives of residents in LTC)	**Non-**<br>trans<br>proac<br>estab<br>to eff<br>maki<br>**Cont**<br>witne<br>deter<br>of-lif<br>Temp<br>clear<br>langu<br>and r
Francisco	2022	Australia	How facilitated case conferences shape interactions and shared decision-making on end-of-life care for LTC residents with advanced dementia	Design: Qualitative Description<br>Data Collection: Transcripts of audio-recorded case conferences	N = 25 case conferences analyzed; residents with advanced dementia, their adult children (proxies), multidisciplinary clinicians (GPs, nurses, PCPCs)	**Lexi**<br>confe<br>medi<br>**Non-**<br>avoid<br>**Cont**<br>balan<br>autho<br>Clini<br>leadi<br>Temp<br>chang

						Orga<br>team<br>orga
Gonella	2022	Italy	Focuses on how contextual factors (political, historical, social, cultural, and family environment) influence the timing, quality, and outcomes of communication in the context of family care conferences for decision-making at the end of life	Design: Integrative case study and situation-specific theory development<br>Data Collection: Residents' clinical records, family caregiver (FC) questionnaires, semi-structured interviews with FCs, NH staff, and NH manager, and in-the-field notes	N = 23 (Pre-family care conference: N = 2 FCs, N = 4 staff<br>Post-family care conference: N =1 1 FCs, N = 4 staff, N = 1 research staff, N = 1 NH manager)	**Lexic**<br>confe<br>careg<br>relati<br>**Non-**<br>in sha<br>trust,<br>resid<br>leadi<br>relati<br>conce<br>**Cont**<br>norm<br>cultu<br>envir<br>quali<br>aroun<br>home
Goossens	2020	Belgium	Shared decision-making in advance care planning for persons with dementia in nursing homes	Design: Cross-sectional observational<br>Data Collection: Comparison of perspectives: nursing home professionals, residents, and external raters on shared decision-making (SDM) levels during advance care planning (ACP) conversations	N = 353 (N = 311 healthcare professionals, N = 42 residents with dementia, family members)	**Lexic**<br>prefe<br>explo<br>guide<br>**Non-**<br>mate<br>poste<br>**Cont**<br>and f<br>Phys<br>ACP<br>infre<br>Temp<br>durin<br>Orga<br>invol<br>backg
Jones	2024	Switzerland	Decision-making practices for residents lacking medical capacity in residential care	Design: Exploratory Qualitative<br>Data Collection: Semi-structured focus groups with nurses and semi-structured interviews with healthcare proxies	N = 54 (N = 23 nurses, N = 13 physicians, N = 18 health proxies)	**Lexic**<br>comm<br>of pr<br>**Non-**<br>**Cont**<br>cultu<br>proxy<br>influe<br>Temp<br>crisis<br>timin<br>Orga<br>pract

Kang	2023	U.S. (9), Europe (5), Taiwan (1), Canada (1)	Timing of Goals of Care (GOC) Discussions in NHs	Design: Systematic Review<br>Data Collection: Systematic review following Preferred Reporting Items for Systematic Reviews and Meta Analyses (PRISMA) guidelines	Included 16 studies including nursing home residents (14 articles) and nursing home personnel (4 articles).	**Lexi**<br>order<br>referr<br>**Cont**<br>deper<br>with<br>capac<br>Temp<br>mont<br>end-c<br>decli<br>Orga<br>struc<br>differ
Kim	2023	South Korea	Factors influencing shared decision-making (SDM) in long-term care facilities	Design: Cross-sectional survey<br>Data Collection: Descriptive survey	N = 280 Staff including nursing staff, social workers, personal care workers	**Lexic**<br>elicit<br>**Non**<br>prese<br>comm<br>**Cont**<br>work<br>deme<br>Temp<br>care<br>Orga<br>clima<br>powe<br>impo
Krishnan	2025	Canada (Manitoba)	Model of nurses' experiences engaging with advance care planning (ACP) in Canadian LTC settings. Explores how nurses facilitate ACP, negotiate goals of care, and ensure comfort for residents during end-of-life and acute medical events.	Design: Constructivist grounded theory<br>Data Collection: Demographic questionnaire, in-depth semi-structured interviews (face-to-face or telephone), field notes, and memos	N = 25 (RNs and LPNs) in 18 LTC facilities	**Lexic**<br>'dow<br>famil<br>decis<br>**Non**<br>and t<br>prere<br>**Cont**<br>such<br>persu<br>advo<br>Temp<br>lettin<br>avoic<br>gradu<br>famil<br>Orga<br>hosp<br>world<br>prior<br>as im
Lemos Dekker	2022	Netherlands	Experiences and expectations of advance care planning for dementia	Design: Naturalistic Interpretive Approach<br>Data Collection: Participant observations, informal conversations, and semi-structured in-	N = 50 (N = 18 people with dementia, N = 22 family caregivers)	**Lexic**<br>tensic<br>main<br>**Non-**<br>patie<br>futur

				depth interviews		**Cont**<br>decis<br>towa<br>patte<br>Temp<br>comp<br>dyna<br>Orga<br>stand<br>emph<br>some
McCarthy	2023	U.S.	How Black and White proxies (decision-makers) experience and perceive ACP and care decisions	Design: Qualitative Description<br>Data Collection: Semi-structured interviews	N = 44 proxies (Black and White family decision-makers of advanced dementia residents)	**Lexic**<br>deme<br>**Non-**<br>influe<br>staff<br>**Cont**<br>and c<br>belie<br>famil<br>regio<br>Temp<br>some<br>Orga<br>inclu<br>comm<br>and c
Mikaelsson	2025	Sweden	Experiences of proactive end-of-life (EoL) conversations in residential care homes	Design: Interpretive Description<br>Data Collection: Semi-structured interviews	N = 19 (N = 11 residents with cognitive decline, N= 8 family members)	**Lexic**<br>conve<br>famil<br>**Non-**<br>cards<br>suppo<br>impa<br>**Cont**<br>invol<br>emph<br>centr<br>Temp<br>proac<br>trajec<br>Orga<br>centr<br>and f<br>imple
Muthui & Paun	2022	Europe (11), U.S. (4), Australia (1)	ACP Process in Nursing Homes	Design: Integrative Literature Review<br>Data Collection: Systematic Review	N =16 studies including nursing home residents with ADRD, family caregivers, and nursing home staff.	**Lexic**<br>treatr<br>discu<br>careg<br>comm<br>satisf<br>**Non-**<br>and e<br>**Cont**<br>careg

						Spati<br>appro<br>sensi<br>Temp<br>trigg<br>due t<br>Orga<br>ACP<br>prese<br>poor
O’Rourke	2022	Canada (Manitoba)	Model providing a comprehensive framework for effective person-centred communication between providers, residents, and families in LTC.	Design: Theoretical model<br>Data Collection: A collective examination and critical analysis of theoretical perspectives used in person-centred communication research to identify gaps and contribute to the expanded model	N = 0 (The paper is primarily a theoretical/model development article and does not present new empirical participants)	**Lexi**<br>adjus<br>resid<br>patro<br>**Non-**<br>provi<br>valid<br>durin<br>roote<br>care<br>**Cont**<br>inclu<br>durin<br>intera<br>cultu<br>cruci
Palan Lopez	2022	U.S.	Nursing home organizational culture and staff perspectives	Design: Interpretive Description<br>Data Collection: Observation, interviews, and document analysis	N = 169 staff (admins, nurses, CNAs, social workers, clinicians, etc.)	**Lexi**<br>proxi<br>maki<br>facili<br>**Non-**<br>staff<br>confi<br>impo<br>beyo<br>**Cont**<br>Blacl<br>favor<br>comr<br>Temp<br>trans<br>Orga<br>staff
Paque	2019	Belgium	Timing of initiation of advance care planning (ACP) after nursing home admission	Design: Prospective observational cohort<br>Data Collection: Structured questionnaire and validated measuring tools (Katz Index of Independence in ADL and MMSE)	N = 741 Newly admitted nursing home residents (N=251 residents with dementia)	**Lexi**<br>care<br>wishe<br>order<br>**Cont**<br>decis<br>deme<br>nurse<br>Initia<br>admi<br>durin<br>year

						Orga<br>proto<br>ACP<br>nomi<br>ACP<br>ACP<br>many
Roach	2023	U.S.	Staff and proxy perspectives on family involvement in decision-making for dementia	Design: Qualitative Description<br>Data Collection: Semi-structured interviews	N = 188 (N =144 nursing home staff, N = 44 proxies)	**Non-**<br>emot<br>**Cont**<br>cause<br>racia<br>relati<br>Temp<br>healt<br>delay<br>Orga<br>cultu<br>staff<br>medi
Saevareid	2019	Norway	Implementation of advance care planning to improve patient participation including those with cognitive impairment	Design: Cluster randomized clinical trial<br>Data Collection: Chart reviews of patient electronic health records (EHRs) at baseline (T0) and after a 12-month intervention period (T1)	N = 454 (N =154 Nursing home patients mostly with cognitive impairment/next of kin, and N = 300 regular staff including nurses and aides)	**Lexi**<br>guide<br>prefe<br>and p<br>**Non-**<br>docu<br>**Cont**<br>suppo<br>healt<br>physi<br>mana<br>Temp<br>patie<br>Orga<br>suppo<br>super<br>appro
Song	2024	U.K. (5), Netherlands (3), Belgium (1)	Optimal timing for initiating advance care planning (ACP) in mild to moderate dementia	Design: Meta-Synthesis<br>Data Collection: Thematic synthesis was used to systematically synthesize qualitative evidence	N = 647 participants across 9 studies (N = 147 people with dementia, N = 210 family caregivers, N = 300 healthcare professionals	**Non-**<br>estab<br>comn<br>conv<br>**Cont**<br>famil<br>profe<br>nurse<br>stage<br>event<br>admi<br>more<br>Orga<br>orgar<br>uncle<br>time<br>profe

Sutherland	2020	Canada (Ontario)	LTC staff perspectives on role in end-of-life decision-making for dementia	Design: Interpretive Description<br>Data Collection: Focus group and semi-structured interviews	N = 21 nursing staff (registered nurses, practical nurses, personal support workers)	**Lexic**<br>chang<br>domi<br>limiti<br>**Non-**<br>emot<br>care a<br>**Cont**<br>limite<br>cultu<br>comm<br>Timi<br>discu<br>time<br>Biom<br>docu<br>dema<br>role l
Sutherland	2019	Canada (Ontario)	Barriers to staff (nurses and support workers) involvement in end-of-life decision-making	Design: Interpretive Description<br>Data Collection: Focus group and semi-structured interviews	N = 21 nursing staff (RNs, RPNs, PSWs)	**Lexic**<br>leads<br>staff<br>death<br>**Non-**<br>routi<br>work<br>**Cont**<br>chall<br>affec<br>confr<br>Temp<br>often<br>occur<br>crisis<br>Orga<br>biom<br>form<br>of-lif<br>interp
Thompson	2020	Canada (Manitoba)	Information and support needs of family caregivers for residents with advancing dementia in nursing homes	Design: Interpretive Description<br>Data Collection: Semi-structured interviews	N = 50 (N = 17 bereaved family caregivers (mainly spouses/adult children), N = 7 palliative care experts, N = 26 nursing home staff (mix of nurses, aides, social worker, therapists)	**Lexic**<br>life, c<br>maki<br>symp<br>**Non-**<br>guilt,<br>**Cont**<br>outsi<br>hesita<br>Spati<br>with<br>conve<br>Temp<br>discu<br>signi<br>or en<br>Orga

						prefe<br>appro<br>atypi
Toles	2018	U.S.	Perceptions of family decision-makers regarding the quality of communication around end-of-life care	Design: Secondary analysis of Cluster Randomized Trial<br>Data Collection: Structured questionnaire (Quality of Communication Questionnaire (QoC) used to measure perceptions of communication quality)	N = 302 Family decision-makers of nursing home residents with advanced dementia	**Lexic**<br>discu<br>items<br>and s<br>**Non-**<br>full a<br>reflec<br>comm<br>clinic<br>**Cont**<br>famil<br>child<br>with<br>(nurs<br>clinic<br>pract<br>Com<br>in lat<br>noted<br>discu<br>Orga<br>comm<br>have<br>role f<br>paym
Unroe	2024	U.S.	NH staff experience with advance care planning (ACP) conversations	Design: Qualitative Description<br>Data Collection: Semi-structured interviews (phone)	N = 14 ACP specialists. Mostly social services staff, female	**Non-**<br>build<br>educa<br>**Cont**<br>activ<br>dyna<br>some<br>Temp<br>and r<br>ongo<br>Orga<br>some<br>cham<br>roles
van der Steen	2025	International (33 countries)	Clinical recommendations on content and process of ACP in dementia	Design: Online Delphi<br>Data Collection: Online Delphi surveys, and interviews with people living with dementia	N = 114 (N = 107 experts from 33 countries, N = 7 persons with dementia)	No f
van Soest-Poortvliet	2015	Netherlands	Factors related to the timing and content of ACP for nursing home patients with dementia	Design: Qualitative Description<br>Data Collection: Interviews	N = 65 (N = 20 family, N = 21 physicians, N = 24 nurses)	**Lexic**<br>decis<br>physi<br>**Cont**

						invol<br>timin<br>reluc<br>varie<br>Temp<br>stay;<br>discu<br>treat<br>trigg<br>physi<br>Orga<br>influ<br>some<br>discu

Structured Search Strategy

Literature Search Terms

**Identifications of Core Elements**	
Participants	Residents living with dementia
Concept/Phenomenon of Interest	Communication in serious illness
Context	Long-term care homes

**Residents living with dementia**	**Communication in serious illness**	**Long-term care homes**
MesH: Dementia<br><br>Keywords:<br>Alzheimer’s and related dementias<br>Dementias:<br>Alzheimer<br>Frontotemporal lobe<br>Lewy Body<br>Vascular<br>Young-onset	Keywords:<br>Advance care planning<br>Care conference<br>End-of-life<br>Goals of care<br>Serious illness<br><br>Within 2 words (adj. 2):<br>Communication<br>Conversation<br>Discussion	MesH: Nursing Homes<br><br>Keywords:<br>Care home<br>Home for the Aged<br>Person care home<br>Residential care home

Inclusion and Exclusion Criteria

Inclusion	Exclusion
**Population**:<br>Residents living with dementia in Long-Term Care homes and their care partners<br>**Age**: Adults<br>**Study purpose**: Any study designed to explore the processes involved in engagement of people living with dementia in LTC homes and their care partners in communication about serious illness related to their health or social care needs.<br>**Study Design**: Published and unpublished primary research studies of any design (qualitative, quantitative, mixed methods, reviews, or theoretical) were included.<br>**Study Content**: Studies had to describe the primary research question, context of the research, study sample, and methods used for data collection and analysis.<br>**Limiter**: English language<br>**Dates**: 10 years (2015-2025)	**Population (wrong study setting)**: Studies not involving people living in Long-Term Care homes (e.g. hospital, alternate level of care, assisted living, or congregate care housing, living at home, or transitioning from home to a care setting).<br>**Population (wrong patient population)**: Studies not about people living with dementia<br>**Intervention (wrong intervention)**: Studies not about the process of serious illness communication (e.g. implementation of interventions, healthcare provider education, clinical pain and symptom management, or broad palliative approach to care).<br>**Study Design**: Study abstracts, protocols, editorials, or opinion pieces.<br>**Study Quality**: Studies that were considered to be of low rigour or relevance, or those that did not provide sufficient descriptive detail and/or theoretical discussion, were excluded.<br><br>In the case of mixed study settings, population, or intervention, people living with dementia in Long-Term Care homes must make up >50% of the participants and >50% of the intervention must include communication about serious illness.

Manuscript ID PCR-26-0022_Revised

Database Search

Database: MEDLINE, Emcare, APA PsycINFO (via Ovid)
Search date: August 8, 2025
Ovid MEDLINE® ALL 1946 to August 8, 2025; APA PsycINFO 1987to August 2025 Week 31; Ovid Emcare 1995 to 2025 Week 31

Search Set	Search Strategy	Results
#1 *Dementia*	exp dementia/ OR (Alzheimer's and related dementias OR Alzheimer OR Frontotemporal lobe OR Lewy Body OR Parkinson OR Vascular Dementia OR Young-onset Dementia).ti,ab.	232170
#2 *Communication in serious illness*	((“Advance care planning” OR “End-of-Life” OR “Goals of care” OR “Serious Illness) adj. 2 (“Communication” OR “Conversation” OR “Discussion”)).ti,ab.	83297
#3 *Setting: Long-Term Care Homes*	Nursing home or (Long-term care or Nursing Homes or Homes for the Aged).ti,ab. or ((“long-term care” or “extended care” or “nursing home” or “nursing facility” or “care home” or “residential care”) and (“resident” or “patients” or “elderly” or “aged” or “long-stay”)).ti,ab.	154046
#4 Combining of major concepts	#1 AND #2 AND #3	341

Database: CINAHL Complete and AgeLine (via EBSCO)
Search date: August 8, 2025

Search Set	Search Strategy	Results
S1 *Dementia*	(MH “Dementia+”) OR (TI dementia OR AB dementia) OR (TI dementias OR AB dementias) OR (TI Alzheimer OR AB Alzheimer) OR (TI alzheimers OR AB alzheimers) OR (TI Alzheimer#s OR AB Alzheimer#s) OR (TI "lewy body" OR AB "lewy body") OR (TI "lewy bodies" OR AB "lewy bodies") OR (TI “Frontotemporal lobe dementia” OR AB “Frontotemporal lobe dementia”) OR (TI “Parkinson dementia” OR AB “Parkinson dementia”) OR (TI “Vascular dementia” OR AB “Vascular dementia”) OR (TI “Young-onset dementia” OR AB “Young-onset dementia”)	23649
S2 *Communication*	(TI “Advance care planning” OR AB “Advance care planning”) OR (TI “End-of-life discussion” OR AB “End-of-life discussion”) (TI “Goals of care” OR AB “Goals of care”) OR (TI “Communication in serious illness” OR “AB “Communication in serious illness”) OR (TI	7166

in serious illness	"Communication about serious illness" OR AB "Communication about serious illness") OR (TI "serious illness conversation" OR AB "Serious illness conversation")	
S3 *Settings: Long-term care homes*	(MH "Long-term care") OR (MH "Nursing Homes") OR (MH "Homes for the Aged") OR ((TI "long-term care" OR AB "long-term care") or (TI "extended care" OR AB "extended care") or (TI "nursing home" OR AB "nursing home") or (TI "nursing facility" OR AB "nursing facility") or (TI "care home" OR AB "care home") OR (TI "residential care" OR AB "residential care)	11080
S4 Combining of major concepts	S1 AND S2 AND S3	72

Deductive Codebook

Lexical Content

**Code name**	**Definition**	**Inclusion Criteria**	**Exclusion Criteria**
Rapport building	Verbal expressions used by residents, care partners, or healthcare providers that establish trust, connection, and empathy in the interaction.	Statements affirming resident preferences, expressing empathy, providing reassurance, or other verbal behaviours that build a trusting interpersonal relationship.	General social greetings or small talk without emotional or relational intent.
Symptom management	Communication involving discussion or explanation of symptom control, treatment options, and management strategies for illness-related symptoms.	Specific references to treatments, medication discussions, prognosis related to symptom control, and reassurance about symptom management efficacy.	Vague or unrelated comments that do not address symptom control.
Illness understanding	Expressions by residents or care partners that reflect their comprehension, acknowledgment, or acceptance of diagnosis and illness severity.	Verbal or written expressions showing awareness or emotional responses to illness status or changes.	Vague or unrelated comments not directly tied to illness comprehension.
Coping with illness	Expressions by residents or care partners reflecting	Statements showing resident's or care partner's emotional	Comments unrelated to illness experience or emotional

	acknowledgement, emotional processing, and adaptation to living with the illness and its challenges.	responses, hopes, fears, or strategies for handling illness challenges.	processing, purely clinical or informational remarks.
Treatment decision-making	Expressed desires or refusals regarding medical treatments, code status, life-prolonging interventions, or comfort care measures.	Verbal or documented treatment choices, including acceptance or refusal of interventions.	General dissatisfaction with care without specific treatment preference.
Supporting unpaid care partners and loved ones	Indicators of care partner involvement, participation, advocacy, or communication dynamics influencing resident care decisions.	Descriptions of care partner/loved ones presence, input, and role in communication processes.	Care partner/loved one absence or irrelevant mentions of care partners.
End-of-life planning	Articulations of life priorities, wishes for quality of life, personal objectives despite illness conditions.	Statements about what matters most, life goals, or hopes for care outcomes.	Statements purely about symptoms or treatments without linking to life goals.

Non-Lexical Content

**Code name**	**Definition**	**Inclusion Criteria**	**Exclusion Criteria**
Tone of voice	Variations in pitch, volume, cadence, and modulation conveying emotions such as empathy, urgency, or reassurance during communication.	Auditory cues that express emotion or attitude, including softness, firmness, tremor, or sharpness in speech.	Neutral or monotone voice without expressive qualities.
Facial expressions	Visible changes in facial muscles that express feelings such as sadness, concern, empathy, or frustration during interactions.	Smiles, frowns, furrowed brows, or eye crinkles indicating emotional states aligned with the conversation.	Neutral facial expressions unrelated to conversation context.
Eye contact	The use, avoidance, or duration of looking directly at or away	Direct gaze maintaining engagement or purposeful	Eyes closed or looking away for reasons unrelated to

	from the conversation partner to convey attention, trust, or discomfort.	avoidance signaling discomfort or disinterest.	communication (e.g., fatigue).
Body language	Posture, gestures, and bodily movements such as leaning forward, crossing arms, or nodding that communicate attitudes or engagement.	Observable physical behaviors aligned with supportive, defensive, or disengaged attitudes during dialogue.	Movements unrelated to communication (e.g., adjusting chair, unrelated distractions).
Silence	Intentional pauses or moments of quiet used to emphasize points, allow reflection, or express empathy during a conversation.	Deliberate breaks in speech that facilitate processing, invite responses, or convey emotions.	Silences caused by interruptions or distractions.
Cadence and conversational rhythm	The flow and pacing of dialogue, including turn-taking, interruptions, and timing that affect communication dynamics.	Smooth and balanced exchanges indicating mutual engagement or disruptions signaling tension or dominance.	Overlapping talk unrelated to communication patterns (unintentional interrupting).

Communication Context

**Code name**	**Definition**	**Inclusion Criteria**	**Exclusion Criteria**
Cultural, family, or relationship factors	Influence of cultural, ethnic, language, religious or social identities on communication preferences, behaviors, and interpretation.	Mention or evidence of cultural practices, language barriers, religious considerations, or family roles affecting communication.	Individual behaviors unrelated to cultural or social identity; generic descriptions.
Participant roles	The dynamic of participant roles, including who leads, contributes, or defers in conversations and how power and authority affect communication.	Documentation of interaction patterns, dominance, partnership, or conflict among communication participants.	Interactions without clear role differentiation or power dynamics.
Physical setting	Environment and spatial arrangement where communication occurs, including privacy, room layout, and noise	Descriptions or observations of physical features that facilitate or hinder communication quality.	Settings unrelated to communication interactions or without impact on communication quality.

	levels that affect interaction dynamics.		
Timing	Timing of communication in relation to illness trajectory, clinical events, or within session moments, influencing the message reception and impact.	Communication occurring at key illness phases (e.g., diagnosis, crisis); timing within sessions affecting pacing.	Timing irrelevant or unknown; unrelated temporal factors.
Organizational and health system factors	Organizational policies, workflows, leadership support, or systemic barriers/facilitators impacting communication practices and uptake.	References to institutional cultures, electronic health record tools, time constraints, or training availability affecting communication.	Factors external to the immediate healthcare system or unrelated institutional influences.

Outcomes

**Code name**	**Definition**	**Inclusion Criteria**	**Exclusion Criteria**
Emotional impact	Immediate emotional or psychological responses (e.g., relief, anxiety, understanding) elicited during or directly after communication.	Observable or reported emotional reactions in residents, care partners, or healthcare providers associated with conversation moments.	General mood or unrelated emotions outside communication
Cognitive reception	Resident or care partner comprehension, recall, and processing of information conveyed during communication.	Demonstrable understanding or confusion related explicitly to communicated content or prognosis.	General cognitive status unrelated to communication.
Perceived communication quality	Participant characterization of communication effectiveness, empathy, and trust gained shortly after the communication event.	Resident/care partner or healthcare provider ratings or expressed perceptions about communication quality in the interaction.	Ratings unrelated to communication specificity or outside the clinical interaction period.
Decisional preparedness	Readiness and confidence of residents or care partners to make informed decisions following communication.	Explicit statements or measures indicating feeling equipped to make decisions about care or treatment.	Decisions or preparedness unrelated to the communication event or absent evidence of readiness.

Behavioural/clinical actions	Observable actions or clinical outcomes that occur as a result of communication, such as treatment decision, documented goals of care, or advanced care plan completion.	Documented follow-up behaviors or clinical changes attributable to prior serious illness communication.	Actions or outcomes unrelated to the communication or occurring too distantly to attribute.
Quality of death	Documentation or indicators reflecting the resident's end-of-life experience quality, encompassing symptom control, alignment with care preferences, and perceived dignity or suffering during dying.	Statements or observations linking care decisions or communication quality to aspects like comfort, symptom control, or goal concordance.	Non-specific references to death or dying without linkage to quality of experience or care preferences.
Goal-concordant care	Evidence that end-of-life care received aligns with the resident's stated values, preferences, or goals of care.	Documentation showing agreement between resident goals and actual care delivered near end-of-life, including adherence to advance directives.	Cases where care delivered is unknown or explicitly discordant with articulated goals.
Grief and Bereavement	Outcomes on care partner emotional, psychological, or practical well-being related to preceding end-of-life decision-making processes or resident's death.	Reports of care partner grief, anxiety, regret, satisfaction, or relief correlated with the decision-making experience or resident outcomes.	General care partner well-being unrelated to end-of-life communications or decision impacts.
Healthcare system	Effects of end-of-life decision processes on healthcare utilization, resource use, transitions, or costs.	Documentation or data showing relationships between decisions and metrics such as emergency department visits, hospital admissions, ICU stays, or medical interventions.	Clinical outcomes unrelated to system-level resource implications.

References

Levoy, K., Tarbi, E. C., & De Santis, J. P. (2020). End-of-life decision making in the context of chronic life-limiting disease: a concept analysis and conceptual model. *Nursing Outlook*, 68(6), 784–807. https://www.doi.org/10.1016/j.outlook.2020.07.008

Tarbi, E. C., Blanch-Hartigan, D., van Vliet, L. M., Gramling, R., Tulsky, J. A., & Sanders, J. J. (2022). Toward a basic science of communication in serious illness. *Patient Education and Counseling, 105*(8), 1951-1960. https://doi.org/10.1016/j.pec.2022.03.019
